# Effects of Three Medicinal Plants Extracts in Experimental Diabetes: Antioxidant Enzymes Activities and Plasma Lipids Profiles inComparison with Metformin

**Published:** 2012

**Authors:** Mohammad Fehresti Sani, Shideh Montasser Kouhsari, Leila Moradabadi

**Affiliations:** *Department of Cellular and Molecular Biology, School of Biology, University College of Sciences, University of Tehran,Tehran, Iran.*

**Keywords:** *Allium ascalonicum*, *Allium sativum*, *Salvia of*fi*cinalis*, Antioxidant enzymes, Plasma lipids

## Abstract

In the present study we aimed to evaluate the effects of methanolic extracts of the bulbs of Garlic (*Allium sativum L., Alliaceae*) and Persian shallot (*Allium ascalonicum L., Alliaceae *) and leaves of Sage (*Salvia officinalis L., Lamiaceae *), ASE, AAE and SOE respectively, on the antioxidant enzymes such as superoxide dismutase (SOD), glutathione peroxidase (GPX) and catalase (CAT) activities and on the levels of plasma lipids profiles such as triglycerides (TG), total cholesterol (TC), high-density lipoproteins (HDL), low-density lipoproteins (LDL) and very low-density lipoproteins (VLDL) in Alloxan diabetic Wistar rats.

In comparison with diabetic control rats in diabetic treated rats, AAE increases the activities of SOD (65%), GPX (43%) and CAT (55%). ASE and SOE increase SOD activity by 60% and 33% respectively. ASE reduces TC (34%), SOE decreases TG (40%) and LDL (30%) and AAE reduces VLDL (24%). Metformin exhibits mild antioxidant and hypolipidemic properties. Results of quantitative phytochemical analysis show that the methanolic garlic and Persian shallot bulbs extracts contain secondary metabolites including alkaloids (3.490% and 3.430%), glycosides (18.023% and 13.301%) and saponins (0.812% and 0.752%). Methanolic sage leaves extract contains flavonoids (1.014%), glycosides (23.142%) and saponins (2.096%). The total phenolic contents of ASE, AAE and SOE were in order 4.273, 3.621 and 6.548 mg GAE/g dry weight (DW).

These results suggest that *Allium sativum*, *Allium ascalonicum *and *Salvia officinalis *are beneficial in the control of diabetes by noticeable antioxidant and hypolipidemic properties.

## Introduction

Oxidative stress, an excessive production of reactive oxygen species (ROS) above the body’s antioxidant capacity, has been implicated in the development of many pathophysiological conditions including diabetes, hypertension, atherosclerosis, cancer and the process of aging (1, 2). Various studies have shown that diabetes mellitus is associated with oxidative stress, leading to an increased production of reactive oxygen species (ROS), including superoxide radical (O_2_•^-^), hydrogen peroxide (H_2_O_2_), and hydroxyl radical (OH•) or reduction of antioxidant defense system (3, 4). The formation of ROS is prevented by an antioxidant system that includs non-enzymatic antioxidants (ascorbic acid, glutathione, tocopherols), enzymes regenerating the reduced forms of antioxidants, and ROS–scavenging enzymes such as superoxide dismutase (SOD), glutathione peroxides (GPX) and catalase (CAT) (5, 6). It is well established that the risk of cardiovascular disease due to atherosclerosis enhance with increasing concentration of total cholesterol and augmented levels of triglycerides in the plasma (7, 8). Patients with type 2 diabetes have a twofold to fourfold excess risk of coronary artery disease as compared to non diabetic patients and many of the primary risk factors for coronary artery disease frequently coexist in these patients (9, 10).

Since the synthetic drugs have undesirable side effects or contraindications, the World Health Organization (WHO) has recommended the evaluation of traditional plants treatments for diabetes (11). Medicinal plants are widely used worldwide to address a variety of health problems. About 25 to 50% of current pharmaceuticals are derived from plants (19, 20). Plants are rich in a wide variety of phytochemical metabolites which are divided into two groups: Primary and Secondary metabolites. Primary metabolites consist of common sugars, amino acids, proteins and chlorophylls, while Secondary metabolites include glycosides, alkaloids, saponins, phenolic compounds, terpenes steroids and anthraquinones (21).

Garlic is known to be effective in decreasing plasma cholesterol and can inhibit LDL oxidation (12). Garlic bulbs active principle agent is allicin, a sulfur-containing compound that with its breakdown products gives to garlic its characteristic odor (13). Also, S-allyl cysteine sulfoxide which is a major antioxidant component of garlic extract has scavenging free radicals property and reduce lipid peroxidation (14). Analysis of Persian shallot extracts has confirmed the presence of flavones and polyphenolic derivatives, suggesting that it also may have antioxidant properties (15, 16). The antioxidant properties of sage extracts is due to its constituents, mainly phenolic compounds such as carnosic, rosmarinic, caffeic and salvianolic acids (17, 18).

In this study we have examined the antioxidant and hypolipidemic effects of methanolic extracts of *Allium sativum*, *Allium ascalonicum *and *Salvia officinalis *in Alloxan induced diabetic rats.

## Experimental

*Chemicals and reagents*

SOD and GPX kits were purchased from Randox (Antrim, UK). The kits for determination of triglyceride (TG), total cholesterol (TC), high-density lipoproteins (HDL) were from ChemEnzyme (Tehran, Iran), Hemoglobin Reagent Set from ZiestChem (Tehran, Iran). All other chemicals and solvents were of the highest commercial grade from Merck (KGaA, Germany) or from Sigma (St. Louis, MO, USA).

*Preparation of methanolic extracts: ASE, AAE and SOE*

Plants materials used in this study consisted of the bulbs of *Allium sativum L. and Allium ascalonicum L*. and the leaves of *Salvia of*fi*cinalis L*. All plants materials were obtained from Tehran province of Iran. They were authenticated by Professor Ahmad Qahraman and voucher specimen as follows: *Allium satium L., *35842, *Allium ascalonicum L.*, 35351 and *Salvia of*fi*cinalis L*., 37221 and were deposited at the herbarium of University of Tehran, Tehran, Iran. About 200gr of dried and ground bulbs of garlic and Persian shallot and leaves of sage were extracted with 300 ml methanol (80%) in a Soxhlet apparatus for 72 h. After extraction, the solvent was filtered and then evaporated by Rotavapor. The percentage yields based on the dried starting materials were 20% for garlic, 17% for Persian shallot and 23% for sage. The powders were stored in the dark at 4°C until being used.

*Preparation of Alloxan-induced diabetic Wistar rats*

Male Wistar rats (*Rattus norvegicus allivias*), weighing 200-250 g were used in this study (Pasteur Institute, Tehran, Iran). Animals were housed six per standard rat cage, in a room with a 12:12 h light/dark cycle and controlled temperature (22 ± 1°C). There were six rats per group in each experiment. The procedures were performed in accordance with institutional guidelines for animal care and use. Diabetes was induced in overnight fasted rats by subcutaneous injection of Alloxan monohydrate (100 mg Kg^-1^ , Sigma, St. Louis, MO, USA), dissolved in citrate buffer (pH = 4.5), according to a previously described method (22, 23).

*Experimental design*

The rats were divided into the six groups, each with six animals. Group I (NC): Normal rats treated with vehicle alone; Group II (DC): Diabetic rats treated with vehicle alone; Group III (ASE+D): Diabetic rats treated with ASE at the dose of 500 mg kg^−1^ BW; Group IV (AAE+D) : Diabetic rats treated with AAE at the dose of 500 mg kg^−1^ BW; Group V (SOE+D): Diabetic rats treated with SOE at the dose of 250 mg Kg^-1^ BW; Group VI (Met+D): Diabetic rats treated with metformin, 100 mg Kg^-1^ BW.

At the end of 21 days of treatment, rats were anesthetized by ether, their blood was collected in both ordinary and EDTA coated tubes for the estimation of plasma lipids levels and superoxide dismutase (SOD), glutathione peroxidase (GPX) and catalase (CAT) activities. Metformin was used as the reference drug.

*Estimation of antioxidant enzymes activities*

The blood of 21 days treated rats were collected in EDTA coated tubes. The activities of SOD (EC: 1.15.1.1) and GPX (EC: 1.11.1.9) were measured using commercial kits. CAT (EC: 1.11.1.6) activity was measured by method of Aebi (24). Activities were expressed as K or U/gHb. The total hemoglobin of samples was measured by a hemoglobin Reagent kit.

*Estimation of blood lipids*

After 21 days of treatment, rats were anesthetized using ether, their blood was collected from heart in ordinary tubes, was allowed to clot, and then, the clotted blood was centrifuged at 2500 rpm for 5 min. Triglycerides, total cholesterol and high-density lipoproteins were estimated using commercial kits. Low-density lipoproteins and very low-density lipoproteins were calculated by formula (25).

*Phytochemical analysis of ASE, AAE and SOE*

Phytochemical analysis was carried out on the plants crude extracts. The total phenolic content for each extract was analyzed with the Folin-Ciocalteau method and was expressed as gallic acid equivalents (GAE) in milligrams per gram dry material (26).The method of Allen’s commercial organic analysis was used for flavonoids determination (27). Alkaloids were determined by method of Henry (28). Glycosides were measured using the method of Analytical Committee of Royal Society of Chemistry. The method of Brunner (29) was used for Saponins determination and the method of strumeyer and malin (30) was used for Tannins.

*Statistical analysis*

All data are presented as means ± S.D. for six rats in each group. Comparisons between groups and between time points were made by one-way analysis of variance (ANOVA) followed by Duncan’s test to analyze the difference. Differences were considered significant when p-values were less than 0.05. All statistical analyses were performed using SPSS (SPSS Inc, Chicago, USA).

## Results and Discussion

*Effects of ASE, AAE and SOE on antioxidant enzymes activities*

The effects of ASE, AAE and SOE are demonstrated in Table 1. As compared with DC group, in ASE+D group, SOD, GPX and CAT activities significantly increased (60%, p < 0.0001), (27%, p < 0.001) and ( 63%, p < 0.043) respectively. In AAE+D group, significant increases were observed in SOD, GPX and CAT activities (65%, p < 0.0001), (43%, p < 0.0001) and (55%, p < 0.043) respectively. In SOE+D group significant raise in SOD (33%, p < 0.0001) and GPX (24%, p < 0.001) activities was observed. Metformin enhanced only slightly the activity of SOD (8% p < 0.043) in comparison with DC group.

**Table 1:** Superoxide dismutase (SOD), glutathione peroxidase (GPX) and catalase (CAT) activities in erythrocytes of ASE, AAE and SOE treated Alloxan-diabetic rats

**Groups**	**Dose (mg/Kg bw)**	**Blood antioxidant enzymes**		
		**SOD (U/g Hb)**	**GPX (U/g Hb)**	**CAT (K/g Hb)**
NC	-	2943 ± 104.4 ^a^	51.31±4.2^a^	1.57 ± 0.6^a^
DC	-	1945.8 ± 21.6	33.11 ± 3.74	0.541 ± 0.011
ASE+D	500	3125.4 ± 54.2 ^a^	42.2 ± 5.21^b^	0.882 ± 0.12 ^c^
AAE+D	500	3214.3 ± 83.2 ^a^	47.5 ± 4.2 ^a^	0.839 ± 0.09 ^c^
SOE+D	250	2591 ± 9.13 ^a^	41.33 ± 2.08 ^b^	0.598 ± 0.04 ^e^
Met+D	100	2118.1 ± 29.7 ^c^	37.31 ± 5.51 ^e^	0.551 ± 0.07 ^e^

NC: normal control, DC: diabetic control, ASE+D: diabetic rats treated with 500 mg kg^−1^ BW of *Allium sativum *bulbs methanolic extracts, AAE+D: diabetic rats treated with 500 mg kg^−1^ BW of *Allium ascalonicum *bulbs methanolic extracts, SOE+D: diabetic rats treated with 250 mg kg^−1^ BW of *Salvia officinalis *leaves methanolic extracts respectively, Met+D: Diabetic rats treated with metformin. Each value is the mean ± SD of ten separate experiments. ^a^ p < 0.0001 vs. DC. ^b^ p < 0.001 vs. DC. ^c^ p < 0.043 vs. DC. ^e^ p > 0.156 vs. DC

*Effects of ASE, AAE and SOE on blood lipids levels*

Table 2 shows the effects of ASE, AAE and SOE on blood lipids levels in Alloxan induced diabetic rats. In ASE+D, AAE+D, SOE+D and Met+D groups, TG significantly reduced (21%, p < 0.001), (35%, p < 0.0001), (40%, p < 0.0001) and (12%, p < 0.041) respectively, as compared with DC group. In comparison with diabetic control group, ASE, AAE and SOE significantly reduce TC (34%, p < 0.0001), (22%, p < 0.001) and (24%, p < 0.001) respectively. LDL reduced mainly in AAE+D group (28%, p < 0.001), SOE+D group (30%, p < 0.001) and ASE+D group (16%, p < 0.041). In AAE+D and SOE+D groups, VLDL reduced significantly (24%, p < 0.001) and (22%, p< 0.001) respectively. In comparison with DC group, HDL shows no difference in plants extracts treated groups (p > 0.156).

**Table 2:** Effect of VFE on serum triglycerides (TG), total cholesterol (TC), high-density lipoprotein (HDL), low-density lipoprotein (LDL) and very low-density lipoprotein (VLDL) in Alloxan-diabetic rats, after 21 days of treatment

Groups	**Dose (mg/Kg bw)**	**Serum lipids (mg/dL)**				
		**TG**	**TC**	**HDL**	**LDL**	**VLDL**
NC	-	85.2 ± 4.8 ^a^	79.5 ± 4.8 ^a^	39.2 ± 5.1 ^a^	21.2 ± 5.6 ^a^	15.3 ± 2.4 ^a^
DC	-	147.6 ± 7.1	118.3 ± 7.8	28.6 ± 5.6	46.9 ± 11.4	26.3 ± 2.1
ASE+D	500	115.4 ± 6.4 ^b^	77 ± 6.2 ^a^	31.4 ± 3.7 ^e^	39.3 ± 7.2 ^c^	23.61 ± 2.7 ^e^
AAE+D	500	95.3 ± 4.6 ^a^	91.3 ± 4.7 ^b^	29.5 ± 5.4 ^e^	33.6 ± 3.8 ^b^	19.8 ± 1.32 ^b^
SOE+D	250	87.33 ± 3.7 ^a^	89.33 ± 5 ^b^	29.6 ± 2.08 ^e^	32.67 ± 3.51 ^b^	20.33 ± 1.54 ^b^
Met+D	100	129.4 ± 5.3 ^c^	106.7 ± 7.7 ^e^	27.1 ± 6.2 ^e^	42.5 ± 8.9 ^e^	24.6 ± 1.3 ^e^

NC: normal control, DC: diabetic control, ASE+D: diabetic rats treated with 500 mg Kg^−1^ BW of *Allium sativum *bulbs methanolic extracts, AAE+D: diabetic rats treated with 500 mg Kg^−1^ BW of *Allium ascalonicum *bulbs methanolic extracts, SOE+D: diabetic rats treated with 250 mg Kg^−1^ BW of *Salvia officinalis *leaves methanolic extracts respectively, Met+D: Diabetic rats treated with metformin. Each value is.the mean ± SD of ten separate experiments. ^a^ p < 0.0001 vs. DC. ^b ^p < 0.001 vs. DC. ^c^ p < 0.043 vs. DC. ^e^ p > 0.156 vs. DC

*Phytochemical analysis of ASE, AAE and SOE*

**Figure 1:**
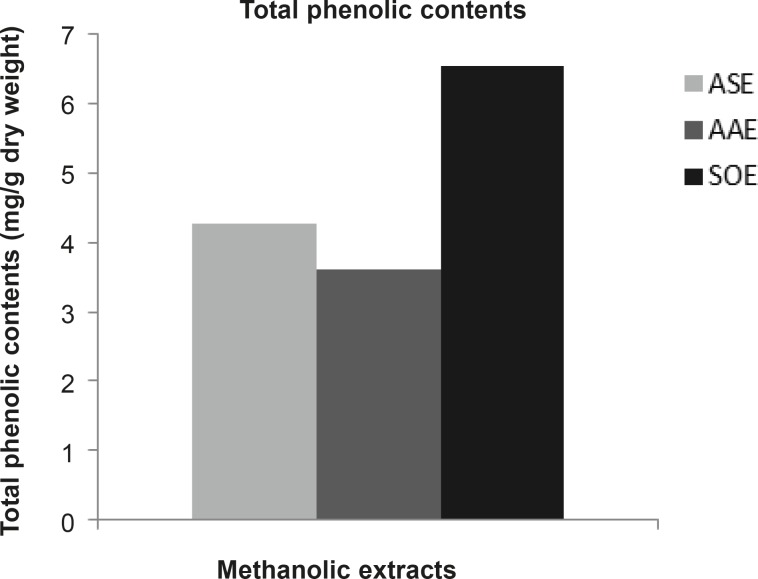
Total phenolic contents of ASE, AAE and SOE by methanolic extraction. The total phenolic contents of ASE, AAE and SOE were in order 4.273, 3.621 and 6.548 mg GAE/g dry weight (DW

The total phenolic contents of ASE, AAE and SOE were 4.273, 3.621 and 6.548 mg GAE/g dry weight (DW) respectively (Figure 1). The results of phytochemical analyses of methanolic extracts of garlic and Persian shallot showed appreciable amount of glycosides, alkaloids and saponins and lesser amounts of tannins and flavonoids (Table 3). Phytochemical analyses of methanolic extracts of sage showed considerable amounts of glycosides, flavonoids and saponins and trace amounts of tannins and alkaloids (Table 3).

**Table 3:** Phytochemical constituents of methanolic extracts off garlic, Persian shallot and salvia

**Phytochemical**	**Garlic (%) + SD**	**Shallot (%)+ SD**	**sage (%) + SD**
Flavonoids	0.049 ± 0.001	0.051 ± 0.001	1.014 ± 0.071
Alkaloids	3.490 ± 0.014	3.410 ± 0.013	0.054 ± 0.001
Saponins	0.812 ± 0.031	0.752 ± 0.011	2.096 ± 0.011
Tannins	0.053 ± 0.001	0.049 ± 0.031	0.812 ± 0.011
Glycosides	18.023 ± 0.089	13.301 ± 0.172	23.142 ± 0.136

Each value is a mean of six determinations

In diabetes mellitus, hyperglycemia can simply inactivate antioxidant enzymes such as SOD, CAT and GPX by glycating these proteins and induces oxidative stress which in turn causes lipid peroxidation (31, 32). Decreased antioxidant enzymes levels and enhanced lipids peroxidation have been well documented in Alloxan-induced diabetes (33-36). In the enzymatic antioxidant defense system, SOD is one of the important enzymes and scavenges the superoxide radicals by converting them to H_2_O_2_ and molecular oxygen. The observed decrease in SOD activity in diabetic control rats could result from inactivation by H_2_O_2_ or by glycosylation of the enzyme, which have been reported to occur in diabetes. CAT and GPX are involved in the elimination of H_2_O_2_ (37, 38).

Based on the findings of the present study, oral administration of ASE, AAE and SOE increased the antioxidant enzymes levels in red blood cells of Alloxan-diabetic rats. ASE and AAE show noticeable increases in SOD, GPX and CAT activities although SOE have milder effects only on SOD and GPX activities. In conclusion, Decreased levels of SOD and CAT in the diabetic state may be due to inactivation caused by reactive oxygen species. In treatment groups the increased CAT activity could be due to higher production of H_2_O_2_. It is possible that CAT activity, which in turn would protect SOD inactivation by H_2_O_2_, and would cause an increase in SOD activity. Increase in SOD activity would protect GPX and CAT against inactivation by superoxide anion (39). Meformin, used as a reference drug, also show slight increase in antioxidant enzymes activities which is due to its hypoglycemic effects.

In accordance with phytochemical screening results of ASE, AAE and SOE, flavonoids, glycosides and phenolic compounds found are suggestive of their antioxidant properties.

In type 2 diabetes, dyslipidemia is characterized through increased levels of serum triglycerides (TG) and reduced levels of serum high density lipoproteins (HDL), while total cholesterol (TC) and low density lipoproteins (LDL) may be either normal or marginally elevated (40). The findings of the [resent study show that in diabetic rats, ASE decreases TG, TC and LDL levels while AAE and SOE decrease TG, TC, LDL and VLDL levels. Metformin decreases s TG levels lightly and shows no effects on other plasma lipids. Saponins found in the plants extracts are suggestive of their antihyperlipidemic properties. It was shown that saponins have hypocholesterolemic activities (41).

In conclusion: The major findings of this study is that, in Alloxan diabetic Wisar rats, *Allium sativum *and *Allium ascalonicum *bulbs and *Salvia of*fi*cinalis *leaves methanolic extracts offer significant protection against oxidative stress and possess capabilities to decrease serum lipids. 

## References

[1] Halliwell B (1994). Free radicals, antioxidants and human disease: cause or consequence?. Lancet.

[2] Chinopoulous V, Adam-Vizi V (2006). Calcium, mitochondria and oxidative stress in neuronal pathology. FEBS J.

[3] Rahimi R, Nikfar S, Larijani B, Abdollahi M (2005). A review on the role of antioxidants in the management of diabetes and its complications. Biomed. Pharmacother.

[4] Vincent AM, Russell JW, Low P, Feldman EL (2004). Oxidative stress in the pathogenesis of diabetic neuropathy. Endocr. Rev.

[5] Zhang XF, Tan BK (2000). Antihyperglycaemic and anti-oxidant properties of Andrographis paniculata in normal and diabetic rats. Clin. Exp. Pharmacol. Physiol.

[6] Lin YF, Tsai HL, Lee YC, Chang SJ (2005). Maternal vitamin E supplementation affects the antioxidant capability and oxidative status of hatching chicks. J. Nutr.

[7] Davy G, Ciabattoni G, Consoli A, Mezzetti A, Falco A, Santarone S, Pennese E, Vitacolonna E, Bucciarelli T, Costantini F, Capani F, Patrono C (1999). In-vivo formation of 8-iso-prostaglandin F2a and platelet activation in diabetes mellitus: effects of improved metabolic control and vitamin E supplementation. Circulation.

[8] Kwiterovich Jr PO (1995). Detection and treatment of elevated blood lipids and other risk factors for coronary artery disease in youth. Ann. NY Acad. Sci.

[9] Marcus AO (2001). Lipid disorders in patients with type 2 diabetes mellitus. Postgrad. Med.

[10] Lee WL, Cheung AM, Cape D, Zinman B (2000). Impact of diabetes on coronary artery disease in women and men. Diabetes Care.

[11] Day C (1998). Traditional plant treatments for diabetes mellitus: pharmaceutical foods. Br. J. Nutr.

[12] Lau BHS (2006). Suppression of LDL oxidation by garlic compounds is a possible mechanism of cardiovascular health benefit. J. Nutr.

[13] Augusti KT (1996). Therapeutic values of onion (Allium cepa L.) and garlic (Allium sativum L.). Indian J. Exp. Biol.

[14] Amagase H, Petesch BL, Matsuura H, Kasuga S, Itakura Y (2001). Intake of garlic and its bioactive components. J. Nutr.

[15] Fattorusso E, Iorizzi M, Lanzotti V, Taglialatela-Scafati O (2002). Chemical composition of shallot (Allium ascalonicum Hort). J. Agric. Food Chem.

[16] Kiviranta J, Huovinen K, Hiltunen R (1988). Variation of phenolic substances in onion. Acta Pharm. Fennica.

[17] Lu YR, Foo LY (2001). Salvianolic acid L, a potent phenolic antioxidant from Salvia officinalis. Tetrahedron Lett..

[18] Wang M, Li J, Rangarajan M, Shao Y, LaVoie EJ, Huang TC, Ho CT (1998). Antioxidative phenolic compounds from sage (Salvia officinalis). J. Agric. Food Chem.

[19] Cowan MM (1999). Plants products as anti-microbial agents. Clin. Microbiol. Rev.

[20] Goh SH, Chuah CH, Mok JSL, Soepadmo E (1995). Malaysian Medicinal Plants for the Treatment of Cardiovascular Diseases.

[21] Habtermariam S, Gray AI, Waterman RG (1993). A new anti-bacterial sesquiterpene from Premma oligotricha. J. Nat. Prod.

[22] Kumar S, Kumar D, Deshmukh RR, Lokhande PD, More SN, Rangari VD (2008). Antidiabetic potential of Phyllanthus reticulatus in alloxan-induced diabetic mice. Fitoterapia.

[23] Kameswara Rao B, Kesavulu MM, Giri R, Appa Rao C (1999). Antidiabetic and hypolipidemic effects of Momordica cymbalaria Hook. fruit powder in alloxan-diabetic rats. J. Ethnopharmacol..

[24] Aebi H, Vergmeyer HU (1974). Catalase in method. Enzymatic Analysis.

[25] Friedwald WT, Levy RI, Fredrickson DS (1972). Estimation of the concentration of LDL-C in plasma without use of the preparative ultracentrifuge. Clin. Chem.

[26] Cliffe S, Fawer MS, Maier G, Takata K, Ritter G (1994). Enzyme assays for the phenolic content of natural juices. J. Agric. Food Chem.

[27] Allen A H (1979). Allen’s commercial Organic Analysis.

[28] Henry TA (1999). The Plant Alkaloids.

[29] Brunner JH (1984). Direct Spectrophotometer determination of saponin. Anal. Chem.

[30] Strumeyer DH, Malin MJ (1975). Condensed tannin in grain sorghum: isolation, fractionation and characterization. J. Agric. Food Chem.

[31] Vincent AM, Russell JW, Low P, Feldman EL (2004). Oxidative stress in the pathogenesis of diabetic neuropathy. Endocr. Rev.

[32] Kaleem M, Asif M, Ahmed QU, Bano B (2006). Antidiabetic and antioxidant activity of Annona squamosa extract in streptozotocin-induced diabetic rats. Singapore Med. J.

[33] Sepici-Dincel A, Acikgoz S, Cevik C, Sengelen M, Yesilada E (2007). Effects of in-vivo antioxidant enzyme activities of myrtle oil in normoglycaemic and alloxan diabetic rabbits. J. Ethnopharmacol.

[34] Oyedemi S, Bradley G, Afolayan A (2011). Antidiabetic Activities of Aqueous Stem Bark Extract of Strychnoshenningsii Gilg in Streptozotocin-nicotinamide Type 2 Diabetic Rats. Iranian J. Pharm. Res.

[35] Mohammadi S, Montasser Kouhsari S, Monavar Feshani A (2010). Antidiabetic properties of the ethanolic extract of Rhus coriaria fruits in rats. DARU.

[36] Monavar Feshani A, Monatasser Kouhsari S, Mohammadi S (2010). Vaccinium arctostaphylos, a common herbal medicine in Iran: Molecular and biochemical study of its antidiabetic effects on Alloxan-diabetic Wistar rats. J. Ethnopharmacol.

[37] Lin YF, Tsai HL, Lee YC, Chang SJ (2005). Maternal vitamin E supplementation affects the antioxidant capability and oxidative status of hatching chicks. J. Nutr.

[38] Soon YY, Tan BK (2002). Evaluation of the hypoglycemic and anti-oxidant activities of Morinda officinalis in streptozotocin-induced diabetic rats. Singapore Med. J.

[39] Blum J, Fridovich I (1985). Inactivation of glutathione peroxidase by superoxide radical. Arch. Biochem. Biophys.

[40] Buse JB, Tan MH, Prince MJ, Erickson PP (2004). The effects of oral antihyperglycaemic medications on serum lipid profiles in patients with type 2 diabetes. Diabetes Obes. Metabolism.

[41] Oakenfull D, Spiller GA (1996). Saponins in the treatment of hypercholesterolemia. Handbook of Lipids in Human Nutrition.

